# Ferritin Genes Overexpression in PBMC and a Rise in Exercise Performance as an Adaptive Response to Ischaemic Preconditioning in Young Men

**DOI:** 10.1155/2019/9576876

**Published:** 2019-04-11

**Authors:** Jan Mieszkowski, Magdalena Kochanowicz, Małgorzata Żychowska, Andrzej Kochanowicz, Agata Grzybkowska, Katarzyna Anczykowska, Piotr Sawicki, Andzelika Borkowska, Bartłomiej Niespodzinski, Jedrzej Antosiewicz

**Affiliations:** ^1^Gdansk University of Physical Education and Sport, Faculty of Physical Education, Department of Biochemistry, Gdansk, Poland; ^2^Gdansk University of Physical Education and Sport, Faculty of Physical Education, Department of Life Sciences, Gdansk, Poland; ^3^Gdansk University of Physical Education and Sport, Faculty of Physical Education, Department of Gymnastics and Dance, Gdansk, Poland; ^4^Medical University of Gdansk, Department of Bioenergetics and Physiology of Exercise, Gdansk, Poland; ^5^Kazimierz Wielki University in Bydgoszcz, Department of Anatomy and Biomechanics, Bydgoszcz, Poland

## Abstract

**Objectives:**

The proposal of this study was to evaluate the effect of acute and ten-day ischaemic preconditioning (IPC) training procedure on the Wingate Anaerobic Test (WAnT), the ferritin H (*FTH*), ferritin L (*FTL*), and transferrin receptor 1 (*TFRC*) mRNA expression in peripheral blood mononuclear cells (PBMC), and anaerobic performance.

**Method:**

34 healthy men volunteers (aged 20.7 ± 1.22 years) participated in the study. The effects of bilateral upper limb IPC and sham controlled condition were assessed in two experimental protocols: (a) the influence of acute (one time) IPC based on an experimental crossover study design and (b) the influence of ten-day IPC training treatment based on a random group assignment. At the beginning and at the end of each experiment upper body WAnT was performed and blood samples were collected to assess gene expression via quantitative PCR (qPCR).

**Results:**

No significant effect of one-time ischaemic preconditioning procedure was observed on upper body WAnT performance. Ten-day IPC training significantly increased upper limbs relative mean power (from 5.29 ± 0.50 to 5.79 ± 0.70 (W/kg), p < 0.05). One-time IPC caused significant decrease in* FTH*,* FTL*, and* TFRC* mRNA levels while 10 days of IPC resulted in significant increase of* FTH *and* FTL* mRNA (from 2 ^∧^254.2 to 2 ^∧^1678.6 (p = 0.01) for* FTH* and 2 ^∧^81.5 to 2 ^∧^923 (p = 0.01) for* FTL*) and decrease in* TFRC *mRNA.

**Conclusions:**

Our findings suggest that ten-day IPC training intervention significantly affects upper limb relative peak power. The observed overexpression of FTH and FTL genes could be associated with adaptation response induced by prolonged IPC.

## 1. Introduction

Ischaemic preconditioning (IPC) is a possible way of improving adaptation to ischaemic stress and increasing skeletal muscle performance. For example, it has been shown that IPC leads to a higher resistance of skeletal muscle to the ischaemic conditions and exercise-induced muscle damage. IPC, when applied in sport, was shown to improve performance [1, 2]. The exact mechanism of how IPC induces these changes is still not well understood. According to Kolh [3] the protective actions of IPC are dependent on the type of organ and applied ischemic preconditioning protocol. Regardless of that, the changes caused by ischaemia start at the molecular level: it changes the activation of some genes, post-transcriptional modification of mRNA, and protein activity in the cells [3, 4]. It has been shown that IPC induces changes in genes expression. The changes in mRNA levels most commonly refer to genes associated with inflammation [4, 5], activation of NF-*κ*B acute-phase response signaling [6], and energy metabolism [7]. In addition, a study on human subjects demonstrated that IPC downregulates leukocyte inflammatory gene expression and suppresses circulating neutrophils activation [8]. Considering that white blood cells (WBC) play a central role in skeletal muscle damage and systemic inflammation, the effects of IPC on these cells are worth exploring.

The inflammation process is strictly connected with iron metabolism [9]. On an organismal level, an increase of proinflammatory cytokines has been shown to stimulate hepcidin biosynthesis [10]. Hepcidin is a hormone which blocks duodenal iron absorption and its liberation from hepatocytes and macrophages to the blood [11]. The biological significance of this is that a limitation of serum iron can decrease oxidative stress and reduce the inflammation process [12]. Intracellular iron can be divided into storage iron that is located in ferritin and the labile iron pool (LIP). Ferritin iron, in contrast to LIP, is metabolically inert and, importantly, it does not participate in iron-dependent formation of reactive oxygen species (ROS) and stimulation of proinflammatory cytokine production [13]. Thus, the level of LIP within the cell will determine the amount of ROS formation and cytokine production. Regulation of LIP in a cell is still not well understood; however it has been shown that upregulation of ferritin H lowers LIP level [14]. In addition, increased ferritin level in cells leads to decreased iron dependent ROS formation [14].

In the current literature there is some data which indicates that an increase in ferritin level in cells plays a protective role against prolonged ischaemia [15, 16]. Effect on ischemia has been done on different types of cells (such like kidney, liver, lung, and skin cells) [4]; however we are not aware of any studies where the responses of ischemia have been evaluated on PBMC human cells. Furthermore, it is hard to establish how exactly IPC influences expression of genes encoding ferritin in humans. Moreover, recently some papers have been published, showing that IPC does not always mediate protection [17, 18]. Thus studying molecular mechanism of ischemic preconditioning could be crucial for the better understanding of adaptive response to ischemia. In this paper we hypothesized that overexpression of* FTH* and* FTL* mRNA in PBMC plays an important role in the adaptation process to IPC and that it will be associated with increased anaerobic performance. For better understanding of changes inside and outside of PBMC, the expression of transferrin 1 receptor (*TFRC*) was also determined. In accordance with the literature [19] we hypothesized that, under ischemic conditions,* TFRC* mRNA level will decrease due to prevention of iron accumulation.

## 2. Methods

### 2.1. Ethics Statement

This study was approved by the Bioethics Committee for Clinical Research at the Regional Medical Chamber in Gdansk and conducted according to the Declaration of Helsinki (KB-24/16). All participants gave their written, informed consent prior to participation and were informed about the possibility of withdrawal at any time for any reason. Prior to their participation, the subjects were informed about the procedures of the study but not about the rationale and aim of the study in order to keep them naive about the potential effect of IPC.

### 2.2. Participants

A group of 34 healthy men volunteered to participate in the study. The baseline physical characteristics of the whole group are shown in Table 1. The subjects were a group of physically active men aged 20.03 ± 0.8 years, declaring regular recreational participation in sports such as running, swimming, and team sports (on average, 2-3 times a week for a duration of 45 min). During the whole experiment period they were not participating in any high-intensity physical activity nor had they been doing any heavy physical workout. The subjects older than 20 years and declaring regular, high-intensity physical activity (physical training) were excluded from the study. The participants did not report any health problems within three months prior to the experiment: no bone or muscle tissue injuries and no intake of drugs. Furthermore, participants had no electrolyte imbalance and had a negative medical history regarding disorders of the respiratory system, nervous system (including epilepsy, mental disorders, and craniocerebral injuries), cardiovascular system (vascular disease, arrhythmia, and high blood pressure—higher than 145/85 mmHg at rest), general infection or physical disability, or any other disease affecting maximal performance as this may affect the efficacy of IPC.

The subjects had visited the laboratory five times and not more often than once a week, including two familiarization sessions to get accustomed to the Wingate Anaerobic Test (WAnT) and IPC protocol.

On the first day, the participants were examined prior to testing by a physician, comprising an assessment of an ECG under resting conditions. On the first visit, the subject's resting blood pressure, resting heart rate, body composition, and height were examined. During all the testing period and a week prior to the testing, the subjects refrained from alcohol, caffeine, guarana, theine, tea, and chocolate as these factors may potentially influence exercise performance. Furthermore, the subjects were asked to adopt a similar eating pattern on all days of the measurements, based on a randomised diet for their age group and physical intensity.

The participants were tested at the same time of day to control for diurnal variation and its impact on exercise performance, while the measurements were performed in a temperature-controlled testing room with the temperature set at 21°C. The testing days were separated by at least seven days interval in order to prevent possible carryover effects of the WAnT and/or IPC. Comparisons of subjects' anthropometric characteristics did not show significant differences in body mass index (BMI) (Table 1).

### 2.3. Experimental Overview

During the experiment the participants were assigned to the two experimental protocols to evaluate (a) the acute effects of IPC and (b) the effects of ten-day IPC training on upper body WAnT and gene expression.

**Figure 1:**
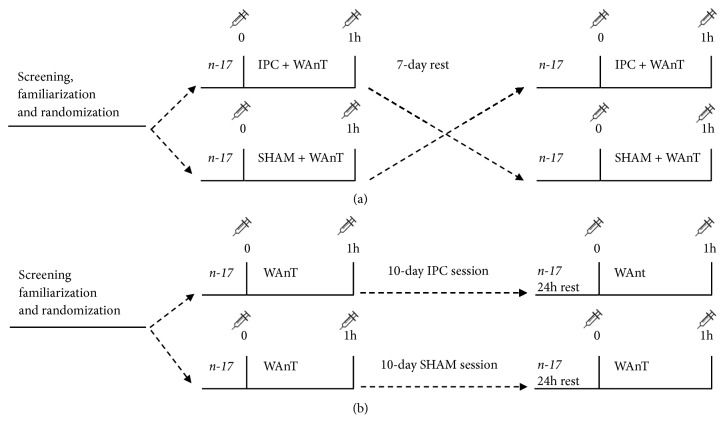
Experimental crossover study design.

Evaluation of the impact of acute IPC was based on an experimental crossover study design (Figure 1(a)). The volunteers were randomly assigned to one of the study groups.

One study group performed IPC and WAnT, and the other group accomplished sham-controlled IPC intervention (SHAM) and WAnT procedure. Both interventions took place half of an hour before the WAnT and were followed by standardized warm-up. Blood samples were collected one hour before and one hour after the WAnT procedure.

After seven days of rest, the participants switched groups and performed analogous research procedures according to the study design.

During the ten-day ischaemic preconditioning training all the volunteers were randomly assigned to one on the study groups: the ten-day training (T-IPC) and the sham-controlled group (CON). During the first week the participants performed only the WAnT procedure and blood samples were collected. The next day, the ten-day experiment began—every day in the ten-day period all the participants had an assigned intervention (IPC or SHAM). 24 hours after the training period ended the WAnT procedure and blood collection was performed once again (Figure 1(b)). In addition, half of the participants (n = 17) (randomly chosen) performed one-time IPC to investigate if the observed changes were an effect of repeated IPC interventions (training), rather than late (24h after) response of single IPC. This procedure was performed after the crossover part of the study. The ten-day ischaemic preconditioning training was performed at the end of the testing period to avoid carryover effect.

All the IPC and SHAM interventions as well as the WAnT and blood sample collection and analysis were performed according to the detailed description in the procedures section below.

### 2.4. Procedures

#### 2.4.1. Anaerobic Components of Fitness: Upper Body Wingate Anaerobic Test

The upper body WAnT was conducted on a hand cycle ergometer (Monark 891E, Langley, WA, USA). The participants sat in a chair fixed to the ground and were advised to keep their feet flat on the ground and to remain seated throughout the WAnT. The seat height and back rest were adjusted so that, with the crank position on the opposite side to the body and the hand grasping the handles, the elbow joint was almost in full extension (140-155°) and the shoulders were in line with the centre of the ergometer's shaft [20].

The familiarization sessions with the WAnT were performed a week before the start of the experiment. A standard resistive load equivalent to 50 g/kg of total body mass was applied for each participant. Before each test the participants completed a warm-up that involved 5 min of arm cranking using a power output of 1 W/kg and a crank rate of 60 rev/min.

During the WAnT each participant was instructed to cycle as fast as possible for 30 s. Verbal encouragement was given to all the participants to maintain their highest possible cadence throughout the WAnT. No further information/feedback (e.g., about the remaining time and achieved power) was provided during the WAnT's trial. The cycle ergometer was connected to a PC to allow data capture via the MCE 5.1 (Staniak 1994, Sport Institute). The following WAnT variables were measured: relative peak power (W/kg) was calculated as the highest single point of power output (recorded at 0.2 s intervals); relative mean power (W/kg) was the average power output during the 30 s test.

#### 2.4.2. Ischaemic Preconditioning Intervention

IPC was performed in the supine position using bilateral arterial occlusion. Occlusion cuffs were positioned proximally around the arm (bilaterally) and inflated to 220 mmHg to block arterial inflow for 5 min, followed by a 5 min deflation (rest). This procedure was repeated 4 times. The procedure was performed during the morning hours, between 8:00 and 10:00, to avoid circadian changes.

The SHAM intervention for IPC was performed under the same conditions as the IPC intervention, but in this case the cuff was inflated to only 20 mmHg, which does not alter the arterial inflow. No additional control test (i.e., without cuff inflation) was performed to keep the participants naive regarding any possible effects of the intervention and, subsequently, to prevent the possibility of a placebo effect. The only information available to the participants during each intervention was the remaining occlusion and deflation time. No further information was provided.

#### 2.4.3. Pain Scale for Ischaemic Preconditioning Intervention

At every IPC intervention, the participants were asked to perceive how ischaemic preconditioning induces pain by marking a number from 0 to 10 on a NRS (Numerical Rating Scale). This procedure evaluated how the IPC procedure is associated with pain symptoms.

#### 2.4.4. Sample Collection and Genes Expression Research Methodology

Blood was collected for genetic analysis in six time points:

(1) Right before and one hour after one-time IPC/SHAM followed by WAnT (Figure 1(a))

(2) Right before and one hour after each WAnT (Figure 1(b)) in training protocol (before the IPC training period and 24h after last training session)

(3) Right before and 24h after single IPC intervention

In order to isolate PBMC, 6 ml of blood was collected directly to BD CPT™ tubes (Becton Dickinson, Franklin Lakes, NJ, USA). After centrifuging, the cells were washed twice using PBS (phosphate-buffered saline) (Sigma-Aldrich, Poland). The remaining PBMC were lysed using Fenozol (A&A Biotechnology, Gdynia, Poland). Further isolation of RNA was carried out according to Chomczynski and Sacchi [21]. The purity and concentration of the isolated RNA were determined by spectrophotometry (Eppendorf BioPhotometer Plus, Eppendorf AG, Hamburg, Germany). From each sample 1000 ng of RNA was used to reverse transcription with oligo dT primers (Transcriptor Kit, Roche, Department Poland).

For the analysis of genes expression, real-time PCR (Applied Biosystem Step ONE, LifeTechnology, Department Poland) was applied in three replicates for each sample using a polymerase (Roche, Poland). The temperature-time profile of the reaction was consistent with the manufacturer's instructions. For each reaction, a melt curve analysis was performed. The Tubulin B (*TUBB*) was used as a housekeeping gene. To amplify the genes, the following primer sequences were applied:

  for TUBB
    R: TGC AGG CAG TCA CAG CTC T
    F: CTA GAA CCT GGG ACC ATG GA
  for FTH
    R: CTG CAG CTT CAT CAG TTT CTC
    F: TCC TAC GTT TAC CTG TCC ATG
  for FTL
    R: CTC GGC CAA TTC GCG GAA
    F: GTC AAT TTG TAC CTG CAG GCC
  For TFRC
    R: AGGCCC ATCTCC TTAACG AG
    F: TGCAGC AGTGAG TCTCTT CA

#### 2.4.5. Statistical Analysis

Descriptive statistics included the mean ± SD for all the measured variables. To investigate the difference of the acute effect (crossover protocol) between IPC and SHAM in the WAnT performance, the unpaired Student t-test was performed. For the ten-day training part of the experiment, a two-way (2 time points × 2 groups) analysis of variance of repeated measures was used to determine the difference between the T-IPC and CON group performances in the WAnT (before and after the training period). Tukey post-hoc tests were applied if significant interaction between main two effects occurred. In addition, the magnitude of the effect size of the differences was estimated according to Cohen's d value [22]. Standardised effects were classified as small (> 0.2), moderate (> 0.5), and large (>0.8). Gene expression data was collected and relative gene expressions were analysed in Excel 2010. In order to calculate the level of gene expression, the method of Schmittgen and Livak [23] was used. To assess statistical significance, the following tests were used: the normality of distribution was checked with the Shapiro-Wilk test and the non-parametric Wilcoxon test was used to compare results before and after testing. All calculations and graphics were performed using GraphPad Prism 6.0 (ftx.pl/program/graphpad-prism). The differences were considered statistically significant differences at a level of p ≤ 0.05.

## 3. Results

### 3.1. Upper Body WAnT Assessment

The characteristics of upper body WAnT performance in all the participants for acute effect of IPC and ten-day IPC training intervention are summarised in Tables 2 and 3, respectively. There was no visible significant effect of one-time IPC on upper body WAnT characteristics expressed as relative peak power (W/kg) and relative mean power (W/kg). However, significantly higher relative peak power after ten-day IPC training was observed in the T-IPC group compared to the CON group. The observed effect was confirmed by the significant Time x Group factor interaction analysis of variance p < 0.05. Scale of pain perception for ischaemic preconditioning intervention indicates that there were no differences between one-time IPC and 10-time IPC (3.25 ± 1.22 vs 2.17 ± 0.89).

### 3.2. Genes Expression

#### 3.2.1. One-Time IPC

**Figure 2:**
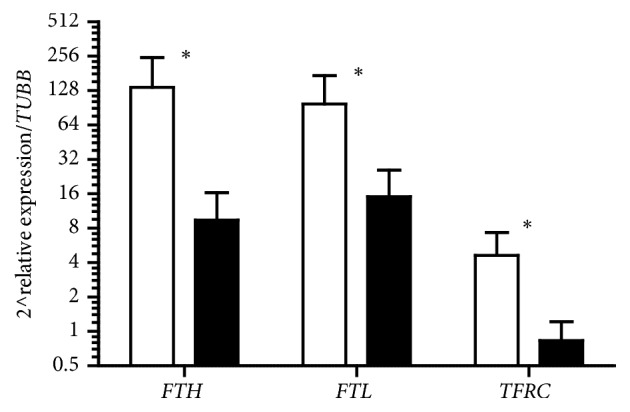
Changes in* FTH*,* FTL*, and* TFRC* mRNA before (white bars) and 24 h after (dark bars) one-time ischaemic preconditioning. *∗* stands for significant difference vs before.

Significant decrease 24h after one-time IPC was observed for* FTH*,* FTL*, and* TFRC *mRNA.* FTH* mRNA mean rest value was 2 ^∧^137 and decreased to 2 ^∧^9.44 (p=0.001) after 24h,* FTL* mRNA declined from 2 ^∧^97.9 to 2 ^∧^15.15 (p=0.003), and* TFRC* mRNA decreased from 2 ^∧^4.63 to 2 ^∧^0.83 (p=0.0004) (Figure 2). Decrease in mRNA levels was observed in all participants.

### 3.3. Ten-Day IPC Training

Changes in* FTH* and* FTL* mRNA levels were evaluated in two groups (CON and T-IPC), while for* TFRC* mRNA only in T-IPC group. Rest values of* FTH* and* FTL* mRNA were comparable in both groups (mean 2 ^∧^238 in CON and 2 ^∧^254.2 in T-IPC for* FTH*, and 2 ^∧^76.2 in CON and 2 ^∧^81.5 in T-IPC for* FTL*). There were also no differences between mean values of tested genes mRNA before one-time and ten-day IPC training.

**Figure 3:**
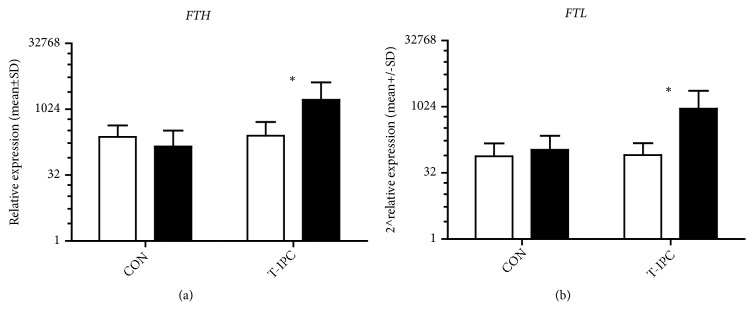
WAnT induced changes in* FTH * and* FTL* mRNA before (white bars) and 24 h after (dark bars) 10 days of IPC training; *∗* stands for significant difference vs before.

After ten days of IPC training, overexpression of* FTH* and* FTL* was observed in T-IPC while in CON these changes were rather small and not statistically significant (Figure 3). In T-IPC, the mean value significantly increased from 2 ^∧^254.2 to 2 ^∧^1678.6 (p = 0.01) for* FTH* and 2 ^∧^81.5 to 2 ^∧^923 (p = 0.01) for* FTL*. Differences in expression between the groups after IPC training were also significant (2 ^∧^143.5 in CON and 2 ^∧^1678.6 in T-IPC (p = 0.009 for* FTH*) and 2 ^∧^106.8 in CON and 2 ^∧^923 in T-IPC (p = 0.01 for* FTL*)).

**Figure 4:**
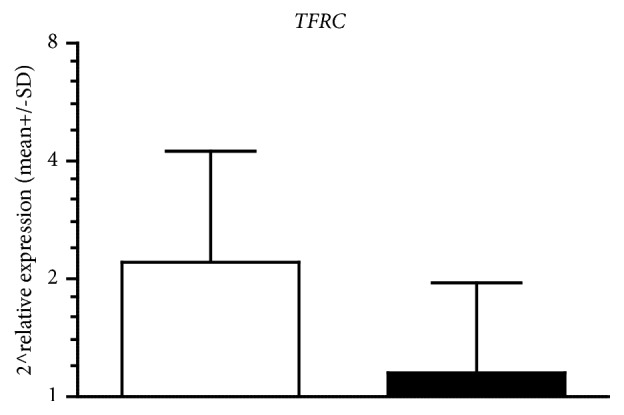
WAnT induced changes in* TFRC* mRNA before (white bars) and 24 h after (dark bars) 10 days of IPC training in T-IPC.

Because of no differences in CON group in* FTH* and* FTL* mRNA, we decided to evaluate changes in TFRC mRNA only in T-IPC. In contrary to ferritins genes, after ten-day IPC training decrease in* TFRC* mRNA levels in T-IPC was observed (from 2 ^∧^3.36 to 2 ^∧^1.15, p=0.054, Figure 4). The data from crossover experiment shows that effect of ten-day IPC training on gene expression was the same in both groups.

## 4. Discussion

To the best of our knowledge this is the first study on human subjects, where IPC effects were investigated in PBMC. We demonstrate that repeated IPC applied on arms leads to a significant improvement in anaerobic performance and induces changes in ferritin and* TFRC* mRNA levels in PBMC.

Interestingly, acute IPC did not alter WAnT performance measured 30 minutes after IPC procedure. Most of the studies showed that IPC induces some adaptive response immediately afterwards and lasts for a few days. Our data is in agreement with a previously published study which showed that IPC did not alter swimming performance 1 h after the IPC; however significant improvement was observed after 2 and 24 h of IPC [2]. However, a study performed on healthy male subjects showed an increase in maximal power output at the exercise test performed 5 min after the IPC procedure [24].

In our study the WAnT was performed on upper limbs and an improvement in relative peak power after ten days of IPC was observed, but there were no differences in relative mean power. WAnT lasted for 30 s and peak power was observed around the sixth second, indicating that this value will be strongly dependent on skeletal muscle ATP and creatine phosphate. In the aforementioned study on swimmers, three 50-m performance trials were conducted [2]. Therefore, this performance is more dependent on anaerobic glycolysis and partially on the mitochondrial system of ATP resynthesis. It can be assumed that in WAnT mean power is mainly dependent on anaerobic glycolysis and partially on aerobic metabolism, but this value did not change either after one-time or after ten sessions of IPC. A similar observation to ours was observed in patients with chronic ischaemic heart failure, where long-term RIPC treatment did not improve cardiopulmonary exercise capacity but significantly increased skeletal muscle power both in the patients and in the healthy control subjects [25].

Significant decrease in* FTH, FTL*, and* TFRC* mRNA was observed 24 h after one-time IPC in PBMC. It is possible that lowered levels of* FTH* and* FTL* mRNA were associated with an increase in translation of ferritin caused by the rise in LIP due to the ferritin degradation. It has been shown that ferritin degradation can be activated by protein kinases such as JNKs (see [13, 16, 26–28]. In addition it has been previously shown that hypoxia may activate JNK [29]; thus it is quite possible that IPC could transiently stimulate ferritin degradation and LIP increase. Decrease in mRNA levels of ferritins after one-time ischaemia could be associated with activity of earlier synthesized mRNA. Expressions of these genes are regulated mainly by activity of mRNA (by the dissociation of IRP1/2 from mRNA 5′UTR IREs). To support this hypothesis* TFRC* mRNA was determined and significant decrease in quantity of these genes copies confirmed our hypothesis. An increase in the LIP stimulates apoferritin synthesis and inhibits* TFRC* expression to minimize the potential of iron toxicity in the cell [19].

Contrary to one-time IPC, upregulation of* FTH* and* FTL* was found in PBMC as an effect of ten-day IPC. The obtained results are hard to discuss for the following reason: there is not much data in the literature in which these genes were investigated in PBMC, while the IPC-mediated protection effect seems to be tissue- and time-specific [30]. Furthermore, PBMC proinflammatory genes expression can easily be induced by oxidative stress appearing in ischaemia and finally caused by activation signaling associated with stress response, i.e., the* HSF-1*,* NF-kB*, and* HIF-1* pathways [31]. Moreover, many other factors—including DAF-16, jun-D, NF-*κ*B, Nrf2, and FOXO3a—were shown to upregulate ferritin gene expression [13, 16, 26–28]. According to Gozzelino and Soares [31], the one-time and prolonged effects of ischaemia can induce different changes in genes expression. This could be the reason for the differences observed in our experiment in ferritin genes expression between one-time IPC and ten-day IPC measured 24 h after.

*FTH* and* FTL* are the Fe-dependent genes. Overexpression of these genes is strongly associated with Fe overload. It is possible that the decrease in* TFRC* mRNA and increase in* FTH *and* FTL* mRNA levels are a part of an adaptive response to IPC.

Free iron is a strong inducer of ferritin gene expression; thus it is possible that in cells where ferritin is upregulated the process was preceded by a rise in free iron. Ferritin is an important part of the cellular antioxidant defence system. Upregulation of ferritin has been shown to lower LIP and increase cell resistance to stress condition [32]. For example, a study performed on animals showed that the protective effects of heart IPC were iron-mediated. Short ischaemic periods applied to the heart, separated by short periods of perfusion, induced an iron-mediated rise in ferritin protein levels. Iron chelators abrogated the protective effects preconditioning and an increase in ferritin [16].

On the other hand, there is literature data which indicates the possibility of heme release from hemoproteins, e.g., haemoglobin under stress condition such as oxidative stress [33]. Some pathogens and microvascular clotting, vasoconstriction, and molecules released in the context of tissue damage can influence lyses and concomitant Hb leakage into plasma [31]. Probably the upregulation of heme oxygenase described in several studies confirms heme degradation under RIPC conditions [4].

In our study, the ratio of* FTH/FTL* in PBMC was similar to the brain or muscle cells [34]—it means higher expression was observed for* FTH.* This proportion indicates tissue-specific manner and is also regulated under stress condition [34]. The function of* FTH* is associated with ferroxidase activity while* FTL* mRNA can be associated with its function: it contributes to the iron incorporation activity due to the more efficient iron nucleation site.

The strong upregulation of* FTH* and* FTL* and decrease in* TFRC* mRNA in PCMB after ten sessions of IPC that was observed by us indicate that some of the preconditioning effects are changes in these genes expressions.

### 4.1. Perspective

Increased ROS formation by leukocytes has been shown to contribute to morbidities including hypertension, heart attack, muscle damage, and many others; however, the role of iron in this process was not studied [35, 36]. The present study shows that repeated IPC applied on arms leads to a significant increase in ferritin genes expression. Considering that ferritin is an important constituent of antioxidant defence [13, 37], it can be assumed that it will lead to higher resistance of PBMC, and possibly other cells, to oxidative stress. It has already been shown that overexpression of ferritin leads to a decrease in the labile iron pool and increased cells resistance to oxidative stress [38]. Free radical-mediated stress has been shown to participate in skeletal muscle fatigue and damage. Whether changes in iron metabolism are responsible for the protective action of IPC requires further study.

### 4.2. Limitations

This is the preliminary study showing that IPC on upper limbs induces changes in three genes involved in iron metabolism. Studies of recent years demonstrate an increasing number of genes involved in iron homeostasis; thus further studies are needed to reveal the effect of IPC on these genes expressions and protein level in PBMC.

## 5. Conclusion

It can be concluded that ten days of IPC significantly increase relative peak power, and this was associated with an overexpression of* FTH *and* FTL* in PMBC and downregulation of* TFRC*. The changes in the overexpression of ferritin genes may be a part of adaptive and protective response to prolonged IPC.

## Figures and Tables

**Table 1:** Acute effect of one-time ischaemic preconditioning (IPC) session on upper body Wingate Anaerobic Test (WAnT) characteristics.

Variables	Overall	T-IPC	CON	(T-IPC vs CON) p value
	Mean ± SD	Mean ± SD	Mean ± SD	
Age (year)	19.90 ± 1.42	19.66 ± 0.61	20.25 ± 1.87	0.26
Body mass (kg)	73.9 ± 6.18	74.9 ± 7.21	73.0 ±5.10	0.40
Height (cm)	177.91 ± 5.90	177.96 ± 5.66	177.18 ± 6.08	0.71
Body mass index (kg/m^2^)	23.51 ± 2.48	23.86 ± 2.38	23.44 ± 2.24	0.69
Body Fat Mass (%)	13.26 ± 2.72	13.89 ± 3.40	12.68 ± 1.86	0.22
Arm lean mass (kg)	3.63 ± 0.38	3.64 ± 0.31	3.62 ± 0.44	0.86

T-IPC: ten-day ischaemic preconditioning training group; CON: sham-controlled group.

**Table 2:** Acute effect of one-time ischaemic preconditioning (IPC) session on upper body Wingate Anaerobic Test (WAnT) characteristics.

WAnT	SHAM	IPC	Δ	p value	Cohen's d
	Mean ± SD	Mean ± SD			
Relative peak power (W/kg)	5.30±0.59	5.42±0.67	0.11±1.03	0.53	0.19
Relative mean power (W/kg)	3.95±0.40	4.02±0.40	0.02±0.59	0.48	0.17

SHAM: sham-controlled ischaemic preconditioning.

**Table 3:** Effect of ten-day ischaemic preconditioning training on upper body Wingate Anaerobic Test characteristics (Mean ± SD).

	Group	Before 10 sessions	After 10 sessions	Δ	Cohen's d
Relative peak power (W/kg)	T-IPC	5.29 ± 0.50	5.79 ± 0.70	0.50 ± 0.58*∗*	0.82
	CON	5.36 ± 0.69	5.46 ± 0.59	0.10 ± 0.46	0.15
Relative mean power (W/kg)	T-IPC	3.88 ± 0.36	4.09 ± 0.41	0.20 ± 0.23	0.54
	CON	3.98 ± 0.49	4.04 ± 0.43	0.06 ± 0.23	0.13

T-IPC: ten-day ischaemic preconditioning training group; CON: sham-controlled ischaemic preconditioning group; *∗*post hoc p < 0.05.

## Data Availability

The data used to support the findings of this study are available from the corresponding author upon request.
